# Effects of Physical Activity and Training Routine on Mental Health During the COVID-19 Pandemic and Curfew

**DOI:** 10.3389/fpsyg.2021.624035

**Published:** 2021-06-03

**Authors:** Jelena Sokić, Stanislava Popov, Bojana M. Dinić, Jovana Rastović

**Affiliations:** ^1^Department of Psychology, Faculty of Sport and Tourism, Educons University, Novi Sad, Serbia; ^2^Department of Psychology, Faculty of Philosophy, University of Novi Sad, Novi Sad, Serbia; ^3^Psychological Centre “Step”, Novi Sad, Serbia

**Keywords:** coronavirus, COVID-19, training routine, athletes, distress, well-being, mental health

## Abstract

This research aimed to explore the effects of physical activity and training routine on mental health during the COVID-19 pandemic and the proclaimed emergency state and curfew. To measure the mental health components of psychological distress and subjective well-being, two studies were conducted on different samples: Study 1 during the beginning of curfew in Serbia (*N* = 678) and Study 2 during the ending phase (*N* = 398). The results of Study 1 showed that elite athletes as well as those with high level of physical activity experienced the lowest distress. Furthermore, effects of the changes in the training routine on distress among physically active individuals depended on the level of sports participation with elite athletes who reduced trainings showing lower anxiety compared to recreational athletes who reduced trainings as well or kept the same training routine. Thus, we could conclude that in the early stage of the pandemic, elite athletes showed better mental health and adaptability to the crisis situation. Results of Study 2 showed that although all the participants had decreased well-being during the curfew, compared to the period before the pandemic and the curfew, there were no differences in well-being between elite and recreational athletes, who had higher well-being compared to non-athletes. However, this effect held both before and during the curfew showing that physically active individuals did not additionally benefit from this decrease compared to the non-active. Furthermore, athletes who became physically inactive showed lower well-being compared to those who kept the same training routine. Thus, in the later stage of the pandemic, prolonged physical inactivity had negative effects on mental health.

## Introduction

Formally declared as a public health emergency of international concern, the outbreak of novel coronavirus (COVID-19) originating in Wuhan, China has spread worldwide and reached the level of a pandemic. To date (April 18th 2021), more than 139 million people have been infected and more than 2.9 million have lost their lives due to COVID-19 (WHO, 2020a). In order to face the spread of coronavirus, authorities in almost all countries worldwide proclaimed health-protection measures. In Serbia, the first case was officially confirmed on March 6th. Due to the rapid spread of coronavirus, a nationwide state of emergency was declared on March 15th. Soon, additional measures were introduced, such as curfew and prohibition of all gatherings in public places and sports courts. The emergency state and lockdown were dismissed on May 6th. Measures such as social distancing remained while prohibition of sports events was canceled and then introduced again.

Many studies have pointed out that the COVID-19 pandemic represents not only a major medical and economic crisis, but also a challenge to mental health. According to WHO (2018), mental health is “more than just the absence of mental disorders or disabilities” such as anxiety and mood disorders for example, and it also refers to “a state of well-being in which the individual realizes his or her own abilities, can cope with the normal stresses of life, can work productively and fruitfully, and is able to make a contribution to his or her community.” Thus, mental health includes both absence or low distress symptoms and indicators of emotional, cognitive, and social well-being. Although health-protection measures help reduce infection rates, changes in the daily routine, social distancing, and reduced physical contact with close people cause increasing mental issues like anxiety and depression (Pappa et al., 2020). Widespread outbreaks of coronavirus are associated with psychological distress and symptoms (for a review see Rajkumar, 2020). A longitudinal study in Germany showed that life satisfaction and positive and negative affect did not change between December 2019 and March 2020, but did decrease in the period of March–May 2020 (Zacher and Rudolph, 2020). The authors explained the surprising decreasing effect on negative affect by a decrease in affective experiences due to demanding situations. However, over time, people adapt to novel circumstances. For example, in one study in Serbia, it was shown that worry, fear, anger, and boredom decreased during the emergency state and curfew, from March 21th till April 24th (Sadiković et al., 2020).

While, up to a certain extent, fear, worry, and stress are considered normal responses to perceived or real threats in the face of uncertainty (WHO, 2020a), prolonged circumstances of chronic stress and quarantine can lead to long-term adverse mental health outcomes. For example, the previous crises and times of uncertainty (e.g., SARS, H1N1 influenza) made us aware of long-term mental health issues (e.g., Liu et al., 2012; Sprang and Silman, 2013), which is aligned with more and more evidence stemming from the current pandemic (e.g., Wang et al., 2020; John et al., 2020). In Serbia, the existing data shows moderate to extreme levels of stress, depression, and anxiety among one third of the participants during the emergency state and the curfew (Popov et al., 2021), as well as a significant proportion of those seeking mental health help due to anxiety (Stašević-Karličić et al., 2020). Moreover, it was shown that a diagnosis of COVID-19 itself, and consequential physical distancing, was associated with feelings of isolation and loneliness (Galea et al., 2020).

Physical activity is most often regarded as an effective coping strategy (Schaal et al., 2011). Unsurprisingly, it is at the top of the list of WHO recommendations, especially when dealing with stressful situations and/or periods, such as COVID-19-induced distress (WHO, 2020b). In this study, we explored the effects of physical activity on mental health during the COVID-19 pandemic.

### Physical Activity and Mental Health

To date, physical activity has been systematically associated with mental health benefits (for a review see White et al., 2017) and reduced symptoms of psychological distress, specifically, depression and anxiety (e.g., Lawlor and Hopker, 2001; Jonsdottir et al., 2010; Ekkekakis, 2015). To name a few, exercise has been found to reduce negative mood and improve self-esteem and cognitive function (Callaghan, 2004), as well as to moderately reduce state and trait anxiety (Stubbs et al., 2017a,b). A mere hour of exercise of any intensity per week has been found to be effective in preventing depression (Harvey et al., 2018). Furthermore, aerobic exercise has emerged as an effective antidepressant intervention (Morres et al., 2019).

That, however, does not mean that individuals highly engaged in sports, i.e., athletes, are immune to mental health problems. As Rice et al. (2016) reported in the first systematic narrative review based on several national studies, generalized anxiety was the highest prevalent disorder found in both male and female athletes (e.g., 8.6% among French athletes, see Schaal et al., 2011; 15% among Australian athletes, see Gulliver et al., 2015). In the case of depression, results suggesting lower rates for the prevalence of depression, but they were somewhat inconsistent (from less than 3% among French, see Schaal et al., 2011; to 15% among all-sports German athletes, see Nixdorf et al., 2016; to 26% in European athletes, see Gouttebarge et al., 2015; and 27.2% in Australia, Gulliver et al., 2015). However, an association was found with several sport-specific factors, such as overtraining, injury, and attribution after failure in a competition (Nixdorf et al., 2016). While frequent anxiety symptoms are not surprising, given the pressures inherent to a competitive environment and high-standard goals (Schaal et al., 2011), lower rates of depression, present to a non-negligible extent, provide further support to the already known effectiveness in the prevention and treatment of depressive symptoms (e.g., Lawlor and Hopker, 2001; Ekkekakis, 2015).

White et al. (2017) went a step further in explaining these inconsistencies, suggesting that, based on their meta-analytic study, engaging in different levels of physical activity is not inevitably associated with better mental health indicators and/or reduced symptoms, but that contextual factors should be regarded as crucial to such a relationship. One of the important contextual factors is the training routine (White et al., 2017). In the light of the pandemic of COVID-19, two groups could provide a better understanding of the effects of physical activity on mental health – those previously engaged in physical activities, but unexpectedly forced to a long-term pause or reduction in their training routines, and those previously less active or inactive, but highly encouraged to introduce physical activity into their daily routine. In order to obtain a more comprehensive insight into the effects of physical activity on mental health, it is important to take into account its contextual counterparts, such as one’s level of sports participation, the intensity of one’s physical activity, as well as the training routine.

### Mental Health and the Level of Sports Participation (Elite vs. Recreational Athletes)

Although physical activity is considered to be undoubtedly beneficial, concerns are rising that high-level athletes (elite or professionals) are as susceptible to certain mental health issues as the general population (Rice et al., 2016). However, the existing literature is often confounded by the fact that many of the studies conducted on the “athletic population” have been based on sports college students and mainly female athletes (e.g., Schaal et al., 2011). Moreover, many studies have failed to include reference groups from recreational exercisers or the general population (Reardon et al., 2019), making it hard to draw clear conclusions.

When considering incidence of mental health issues, Gerber et al. (2011) showed that competing at an elite level neither contributes to the risk of depressive symptoms or additional distress nor can it be considered as a protective factor (Gerber et al., 2011). Similar, Castillo et al. (2010) suggested that high-level competition does not affect the risk of developing psychopathological symptoms. However, they found that perception of the situation matters and that higher levels of competition perceived by athletes is to be associated with a lower incidence of symptoms and a higher level of well-being. Additionally, Shirvani et al. (2015) showed that semi-professional athletes were prone to more adaptive emotion regulation strategies, compared to amateur athletes. In contrast, Peluso and Andrade (2005) showed that more intense physical activity performed at professional/elite levels can compromise mental health.

However, when considering specific stressors, the results seem to differ and highlight the presence of mental issues among elite athletes. In fact, Gulliver et al. (2015), for example, found that in increased stress situations (e.g., overtraining or injuries), elite athletes were more likely to experience symptoms of mental disorders than the general population, but no comparisons were made with recreational exercisers. Similarly, Oztekin et al. (2008) reported that professional athletes scored higher on depression than amateurs before and after injury-related surgery. Apart from injury, retirement, aging and competitive failure were found to precipitate depression (Reardon and Factor, 2010). Some authors (Cresswell and Eklund, 2007; Hughes and Leavey, 2012) explained that the elite sport environment limits the ways of shaping one’s identity in the way that it generates an “identity foreclosure” (Hughes and Leavey, 2012, p. 95). Therefore, when the athletic identity is threatened to be removed, e.g., due to injury or retirement, there is a higher risk for elite athletes to experience psychological distress and other mental health issues. Together, these results may suggest that, while elite levels of sport participation are not a default risk factor for mental health problems and that prolonged exposure to pressures might lead elite athletes to adapt and become better at emotion regulation, the context of bigger or unexpected stressors might put elite athletes at a higher risk of psychological distress. This could reflect greatly on athletes’ ways of coping with the unpredictable situation caused by the current COVID-19 pandemic.

Having in mind the prevalence of mental health issues related to sport-specific stresses and challenges among elite athletes, it is justified to wonder whether exercise is only beneficial up to a certain point and whether elite athletes are actually more susceptible to mental health problems due to chronic exposure to stresses and pressures (e.g., Peluso and Andrade, 2005; Cresswell and Eklund, 2007; Hughes and Leavey, 2012). In other words, the beneficial psychological effects of physical activity may be limited to individuals at the recreational (or sedentary) level, while in the context of elite sports, those unique stressors might have detrimental effects. Conversely, another possibility could be considered. As elite athletes are frequently characterized as being able to adapt and thrive under pressure (Connaughton et al., 2008), prolonged exposure to pressure could lead to better adaptations in a unique stress situation, such as the current COVID-19 curfew.

### Mental Health and the Intensity of Physical Activity

As mentioned above, many studies focusing on mental health have failed to differentiate between elite and recreational athletes, resulting in inconsistent results and limited conclusions about the prevalence of psychopathological symptoms. Yet, another confounding factor is the intensity of physical activity related to specific sports. For example, although sports such as archery require the same level of professionalism and commitment as long-distance running, the intensity of physical activity and the demands for high-level performance are very different. The importance of the intensity of physical activity is particularly visible in WHO (2020b) guidelines for the optimal effects on well-being, suggesting 50–300 min of moderate-intensity or 75–150 min of vigorous-intensity physical activity per week (Bull et al., 2020).

When it comes to the general population, a few studies that have examined the relationship between the intensity of physical activity and mental health have offered mostly inconsistent results. One group of studies showed benefits of high or vigorous physical activity. For example, Steptoe and Butler (1996) found vigorous activity to be positively related to emotional well-being regardless of sex, social class, and health status. Costigan et al. (2019) found that light and moderate physical activity was not associated with well-being, but that vigorous physical activity was associated with more positive affect among adolescents. In the context of COVID-19, results of a recent study showed that participants performing high physical activity showed lower values of state anxiety compared to participants performing moderate or low physical activity (Frontini et al., 2021).

However, other groups of studies found that moderate-intensity activity was the most beneficial activity level for improving well-being (Netz et al., 2005), that it significantly added to happiness (Downward and Dawson, 2016), that it could reduce short-term physiological reactions to brief psycho-social stressors (Taylor, 2000) and that it was positively associated with subjective well-being compared to high-intensity physical activity which showed a negative effect (Wicker and Frick, 2015). There are also results showing that light-intensity physical activity produced the highest overall subjective well-being, but moderate-intensity was associated with the lowest overall value of subjective well-being (Downward and Dawson, 2016). It should be noted that Asztalos et al. (2012) found negligible differences in perceived stress and emotional distress among individuals engaging in different types of sports regarding intensity, suggesting that “one-activity-fits-all” recommendations are inappropriate.

It is worth noting that methodology-wise, the general limitations of most of these studies are the use of a single domain of mental health (e.g., happiness) and the lack of comparison between all possible intensities of physical activity (i.e., light, moderate, and vigorous, see Panza et al., 2019). Furthermore, previous studies have mostly relied on heart rate or accelerometry, which has been criticized for failing to distinguish between different types of physical activity behaviors (Timperio et al., 2004). More importantly, there seems to be no study addressing this issue in the context of high-level sports participation or distinguishing elite from recreational athletes.

### The Current Study

Despite the fact that engagement in physical activity has been related to good mental health (e.g., White et al., 2017), the effects of physical activity and exercise routine on mental health in a crisis such as a pandemic have been found to be inconsistent. For example, Maugeri et al. (2020) found that 1 month into the COVID-19 quarantine in Italy, physical activity decreased and the reduction in physical activity had a profoundly negative impact on psychological health and well-being. However, Zhang et al. (2020) showed that the relation between life satisfaction and the severity of COVID-19 in one’s local community was surprisingly negative in people who exercised more during the outbreak (more than 2.5 h per day). On the other hand, the relation was not significant for people who exercised between 1 and 2.5 h and it was positive for people who exercised up to 0.5 h.

The COVID-19 pandemic and the introduced compliance measures such as a lockdown or curfew are known to be stressful for the general population (e.g., Rajkumar, 2020; Zacher and Rudolph, 2020). The existing literature has suggested that athletes are at a comparable risk for clinically significant psychological distress as the general population (Putukian, 2016). Moreover, there have been indications that it could be even more detrimental for athletes, as the curfew forced many to give up their daily routines. Many athletes had to suddenly halt their training and competition participation.

Due to these inconsistent results, the goal of this research was to further explore how this uncertain and undoubtedly stressful situation caused by the COVID-19 pandemic could impact the mental health of individuals at different levels of sports participation and physical engagement. This study aimed to determine whether the pandemic and the introduced compliance measures put additional pressure on those engaging in high-level sports (i.e., elite athletes), relative to those less involved (i.e., recreational athletes or non-athletes), or whether those moderately or highly involved in physical activity could be more resilient in the time of a pandemic. Thus, the aim of this research was to explore the effects of physical activity and training routine on mental health during the coronavirus pandemic and the proclaimed emergency state and curfew in Serbia. In order to add an additional value, we sought to address the above-mentioned methodological issues. We conducted two studies in which different mental health domains were assessed. Further, we employed a more comprehensive approach by taking into account the intensity of physical activity besides sports participation, and determined the mental health before and during the emergency state and curfew.

## Study 1

The aim of Study 1 was to explore the effects of physical activity on psychological distress during the early stage of curfew. Physical activity was assessed via two variables: (1) as a broad measure of physical activity regarding sports participation with a differentiation between non-athlete, recreational athlete, and elite athlete participants, as in the majority of research, and (2) as the intensity of physical activity (sedentary, moderate, and highly active). Additionally, we tested the differences in psychological distress depending on changes in the training routine during the curfew among those who previously had a training routine, i.e., recreational and elite athletes. It could be assumed that athletes are more adaptive to the context of stress, since the majority of studies have revealed higher cognitive resources and extensive experience of athletes (especially professional athletes) in coping with anxiety contexts like competitions (Belinchón-deMiguel et al., 2019). Thus, we expected that physically active participants (both recreational and elite athletes) would have lower distress during the curfew compared to non-athletes (H1). Due to the conflicting results regarding mental health differences between elite and recreational athletes (e.g., Peluso and Andrade, 2005; Gerber et al., 2011), and the specific global effects of the pandemic as a stressful event for everyone (e.g., Rajkumar, 2020), we did not expect significant differences between elite and recreational athletes. In line with the previous research (Frontini et al., 2021), we further expected that those who performed higher intensity of physical activity will experience less distress during curfew compared to those who performed moderate or low intensity of physical activity (H2). Finally, we hypothesized that both recreational and elite athletes who did not change their training routine will experience lower distress compared to those who trained less or became inactive (H3). Since we do not expect differences between the elite and recreational athletes, we also do not expect that level of sports participation moderate the effects of training routine on distress.

### Method

#### Participants and Procedure

In total, 678 individuals (66.2% females, *M*age = 35.85, *SD* age = 12.45, age range 18–78) participated in the study. The sample consisted of 20.5% (139) non-athletes, 64% (434) recreational athletes, and 15.5% (105) elite athletes. These groups were differentiated based on self-report question. Based on the self-report question, among the physically active, three groups were formed regarding training routine during the pandemic and the curfew: those who became inactive (79 or 14.7%), those who reduced their trainings (399 or 74%) and those who kept the same training routine (61 or 11.3%).

There were sex differences in the level of participation [χ^(2)^ = 63.63, *p* < 0.001] and the intensity of physical activity [χ^(2)^ = 21.13, *p* < 0.001], with males more often belonging to the group of elite athletes and being more physically active. Additionally, there were also age differences in the level of sport participation with elite athletes were the youngest [*F*(2,675) = 14.60, *p* < 0.001], while there were no significant age differences regarding the intensity of physical activity [*F*(2,669) = 12.24, *p* > 0.05]. In preliminary analysis, statistically controlled sex and age did not have any influence to the obtained effects, thus they are not included as control variables.

The measures were administered online, via social networks, local online magazines, and sports-related websites. Data were collected during the period between March 31st (3 weeks after the declaration of a nationwide state of emergency and curfew) and May 6th (when the emergency state and curfew officially ended), while the majority of the sample (74%) fill the measures until April 19st (fifth week of emergency state and curfew). The study was approved by the Ethical Committee of Faculty of Sport and Tourism.

This study is a part of a larger project which includes other measures, but the participants completed measures that refer to the current state first and then continued to the other trait-like measures. The same sample was used in Popov et al. (2021), although the aim of the study was different.

#### Measures

The Depression, Anxiety and Stress Scale (DASS-21; Lovibond and Lovibond, 1995; the Serbian adaptation was available at the official website^1^, and for further information about the validation see Jovanovic̀ et al., 2014). The instrument consists of three subscales: (1) depression, which assesses the degree of dysphoria, hopelessness, low-self-esteem, anhedonia, and passivity (α = 0.87); (2) anxiety, which refers to the subjective feeling of situational anxiety, autonomic arousal, and skeletal muscle tension (α = 0.87); and (3) stress, which measures difficulties in relaxing, nervous arousal, and the tendency to be easily agitated, irritable, and upset (α = 0.90). The participants were asked to report how they felt during the previous week, due to the emergency state and curfew-related changes in daily functioning.

The Godin Leisure-Time Exercise Questionnaire (GLTEQ; Godin and Shephard, 1985; adapted into Serbian language by Pinc̀ir et al., 2020) is a self-report measure of weekly physical activity. The original version measures the level of physical engagement during the previous week, during one’s free time, in duration exceeding 15 min. For the purpose of this study, we adapted the instruction and asked for the level of intensity *before* the emergency state. The measure differentiates between three levels of physical activity: strenuous/vigorous (e.g., running, squash or roller skating), moderate (e.g., fast walking, easy bicycling or tennis), and mild (e.g., yoga, easy walking or archery). The weekly frequencies of mild activities are multiplied by three, moderate by five, and strenuous activity by nine three metabolic equivalents. The total sum of all these activities forms a leisure activity score (fewer than 14 units – sedentary, 14–23 moderately active, and 24 or more – highly/vigorously active).

Additional multiple-choice question was added in order to assess the changes in the routines of physical activity due to the curfew (“*Since the curfew was imposed, how has your training routine been changed*?”).

### Results

Firstly, comparison between frequencies of the level of participation and the intensity of physical activity was calculated (Table 1). Results showed significant differences [χ^2(4)^ = 235.48, *p* < 0.001] with vigorously active participants including 100% of elite athletes, followed by recreational athletes and non-athletes, while sedentary participants included almost a half of non-athletes. Since there are no moderately active and sedentary participants among elite athletes, a fully interaction model in two-way ANOVA with level of sport participation and intensity of physical activity as between-subject factors is not possible, thus we only reported separated one-way ANOVAs.

**TABLE 1:** Cross-tabulation of the level of sport participation and the intensity of physical activity.

	**Intensity of physical activity**			
**Level of sport participation**	**Highly active**	**Moderately active**	**Sedentary**	**Total**
Elite athletes	105 (100%)	0	0	105 (100%)
Recreational athletes	310 (72.3%)	72 (16.8%)	47 (11%)	429 (100%)
Non-athletes	26 (18.8%)	32 (23.2%)	80 (58%)	138 (100%)
Total	441	104	127	672

*Six participants did not complete the Godin Leisure-Time Exercise Questionnaire (GLTEQ), and thus, instead of 678, there were 672 participants in total included in the analysis based on the intensity of physical activity.*

We obtained significant differences between the groups based on the level of sport participation on all three dimensions of psychological distress (Table 2). *Post hoc* Bonferroni tests revealed that the lowest levels of anxiety, depression, and stress were reported by elite athletes, followed by recreational athletes, while the highest scores were reported by non-athletes.

**TABLE 2:** Results of one-way ANOVAs: differences in psychological distress during the curfew between non-athletes, recreational athletes, and elite athletes (*N* = 678).

**Psychological distress**	**1 = Non-athletes (139)**	**2 = Recreational athletes (434)**	**3 = Elite athletes (105)**	***F*(2,675)**	**η_p_^2^**	***Post hoc***
		
	***M*(*SD*)**	***M*(*SD*)**	***M*(*SD*)**			
Anxiety	4.29 (5.16)	3.07 (4.35)	1.72 (3.69)	10.03***	0.029	1 > 2 > 3
Depression	5.58 (5.61)	3.97 (4.68)	2.55 (4.05)	12.20***	0.035	1 > 2 > 3
Stress	9.07 (5.75)	7.43 (5.63)	4.96 (4.66)	16.64***	0.047	1 > 2 > 3

***p < 0.001.

We further inspected the differences in psychological distress by taking into account the level of intensity of physical activity our participants engaged in before the curfew. Again, significant effects of the level of physical intensity were obtained on all dimensions of psychological distress (Table 3). *Post hoc* Bonferroni tests showed that vigorously active individuals, compared to the moderately active, reported significantly lower scores on anxiety and depression. Furthermore, vigorously active individuals reported significantly lower scores on stress compared to both moderately active and sedentary participants.

**TABLE 3:** Results of one-way ANOVAs: differences in psychological distress during the curfew regarding the intensity of physical activity before the curfew (*N* = 672).

**Psychological distress**	**1 = Sedentary (127)**	**2 = Moderately active (104)**	**3 = Highly active (441)**	***F*(2,675)**	**η_p_^2^**	***Post hoc***
		
	***M*(*SD*)**	***M*(*SD*)**	***M*(*SD*)**			
Anxiety	3.65 (4.54)	4.14 (5.16)	2.73 (4.28)	5.24**	0.015	3 < 2
Depression	4.76 (5.13)	5.03 (5.46)	3.68 (4.63)	4.74**	0.014	3 < 2
Stress	8.72 (5.58)	8.60 (5.73)	6.73 (5.56)	9.16***	0.027	3 < 1, 2

***p < 0.001, **p < 0.01.

**FIGURE 1:**
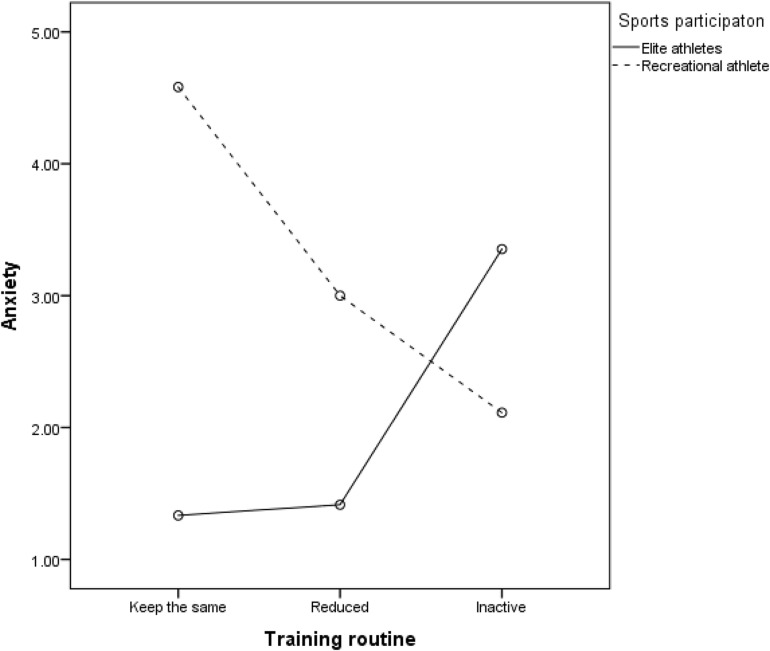
The interaction between sport participation and the training routine during the curfew on anxiety among physically active participants (elite and recreational athletes, *N* = 539).

Finally, we tested the differences in psychological distress by taking into account the changes in the training routine during the curfew among physically active, i.e., elite and recreational athletes, since only these two groups had the training routine in the past. We conducted two-way ANOVAs with training routine and level of sports participation as factors and distress scales as dependent variables in each analysis. Results showed a significant main effect of level of sport participation on stress (Table 4), with elite athletes showing lower scores (*M* = 4.96, *SD* = 4.66) compared to recreational athletes (*M* = 7.43, *SD* = 5.63), which had also been obtained in the previous analysis. Training routine did not show a significant main effect on either distress scale. However, there was marginally significant interaction effect on anxiety. *Post hoc* Bonferroni tests showed that there were no significant differences among elite athletes regarding anxiety level that depends on the training routine. However, recreational athletes who kept the same training routine showed higher anxiety compared to those who reduced trainings (Figure 1). Furthermore, elite athletes who reduced trainings showed lower anxiety compared to recreational athletes who also reduced or kept the same training routine during the curfew.

**TABLE 4:** Results of two-way ANOVAs: differences in psychological distress regarding the level of sport participation (recreational and elite athletes) and changes in the training routine during the curfew (*N* = 539).

	**Anxiety**		**Depression**		**Stress**	
**Effects**	***F***	**η_p_^2^**	***F***	**η_p_^2^**	***F***	**η_p_^2^**
Level of sport participation	2.67	0.005	0.96	0.002	7.36**	0.014
Training routine	0.60	0.002	1.59	0.006	0.73	0.003
Interaction	3.17*	0.012	0.56	0.002	0.06	0.000
Physical activity	1.40	0.005	0.98	0.004	3.37*	0.013
Training routine	3.45*	0.013	1.37	0.005	0.47	0.002
Interaction	0.40	0.003	0.77	0.006	1.05	0.008

df_*bg*_ for level of sport participation was 1, for training routine and interaction was 2, and df_*error*_ = 533; df_*bg*_ for physical activity was 2, for training routine and interaction was 2, and df_*error*_ = 525; **p < 0.01, *p < 0.05.

Additionally, we tested effects of physical activity and training routine on distress scales. Results showed that physical activity had the main effect on stress (Table 4). *Post hoc* Bonferroni test showed that vigorously active individuals (*M* = 6.56, *SD* = 5.45) had lower stress scores compared to sedentary individuals (*M* = 8.83, *SD* = 5.67). There was also the main effect of training routine on anxiety, with those who kept the same training routine (*M* = 4.20, *SD* = 5.02) showing higher anxiety compared to those who became inactive (*M* = 2.38, *SD* = 3.56) or reduced their trainings (*M* = 2.69, *SD* = 4.25). However, there was no significant interaction.

In sum, we partly support H1 by showing that physically active participants had lower distress compared to physically inactive participants. However, there are also differences between elite and recreational athletes, with elite athletes experiencing the lowest distress, which was not in line with H1 stating that elite and recreational athletes would not differ in distress. Furthermore, we support H2 by showing that participants with vigorous physical activity showed the lowest scores on distress scales compared to the moderately active and/or sedentary. Contrary to our expectations (H3), we found the effects of the training routine on anxiety to be dependent on the level of sports participation. Specifically, elite athletes who reduced their trainings showed lower levels of anxiety compared to recreational athletes who also reduced or kept the same training routine.

## Study 2

The aim of Study 2 was to further explore the differences in well-being among non-athletes, recreational athletes, and elite athletes during the ending stage of the curfew. Since the previous study did not include measures of mental health domains before the pandemic, in this study, we asked the participants to fill out the measures by following two instructions: (1) before the pandemic and the emergency state and (2) in the previous week, during the emergency state and curfew. Firstly, based on the previous research (e.g., Zacher and Rudolph, 2020), we expected that well-being decreases among all the participants during the curfew (H1). However, we expected that physically active (both recreational and elite athletes) would have better well-being both before and during the curfew compared to physically inactive, i.e., non-athletes (H2). In addition, we expected that decrease in well-being would be smaller among physically active (H3). Thus, due to the better mental health among physically active individuals (e.g., White et al., 2017), we could expect that being physically active provides benefits for mental health in crises and stress situations. Further, we tested the differences in well-being depending on the changes in the training routine among the physically active. We assumed that both recreational and elite athletes who did not change their training routine during the curfew would show better well-being compared to those who became inactive or changed the training routine, i.e., reduced or increased trainings (H4).

### Method

#### Participants and Procedure

The sample included 441 participants, 43 (9.75%) of whom were in quarantine and thus excluded from the final sample. Among 398 participants, 61.1% women, age range 18–73 (*M* = 34.83, *SD* = 10.21), 103 (25.9%) were non-athletes, 180 (45.2%) were recreational athletes, and 115 (28.9%) were elite athletes. The differentiation between recreational and elite athletes was based on questions about sport type and participation in competitions during the previous year. Thus, those who reported that they participated in a competition and engaged in types of sports such as collective and endurance sports in contrast to recreational types (e.g., fitness, gym, recreational running and cycling) were identified as elite athletes, while the rest were categorized as recreational athletes. Elite athletes had more participation in competitions during the previous year [*t*(293) = –4.58, *p* < 0.001, *M*elite = 14.37, *SD*elite = 29.72, *M*recreational = 2.26, *SD*recreational = 15.51], they have been involved in physical activity for a longer period [*t*(286) = –2.16, *p* = 0.032, *M*elite = 12.68 years, *SD*elite = 10.74, *M*recreational = 9.98 years, *SD*recreational = 9.98], and they had more frequent training routine before the curfew compared to recreational athletes [*t*(293) = –3.26, *p* = 0.001, *M*elite = 3.26 on a scale from 1 to 5, *SD*elite = 0.77, *M*recreational = 2.94, *SD*recreational = 0.86]. Based on the reported frequency of the training routine by answering the two questions – before and during the emergency state, four groups were formed: those who became inactive, those who reduced trainings, those who kept the same routine, and those who increased trainings (see Table 5). Since sports fields, swimming pools, and other sports areas were closed, we asked the participants to report their training routines during the curfew, including sports other than their primary type.

**TABLE 5:** Results of one-way ANOVAs: differences in well-being regarding the changes in the training routine during the curfew among recreational and elite athletes (*N* = 295).

**Well-being**	**1 = Inactive (43)**	**2 = Reduced trainings (51)**	**3 = Keep the same training routine (143)**	**4 = Increased trainings (58)**	***F*(3,291)**	**η_p_^2^**	***Post hoc***
Satisfaction with life	3.91 (1.51)	4.06 (1.36)	4.13 (1.29)	4.23 (1.52)	0.46	0.005	–
Joviality	2.52 (0.94)	2.95 (0.94)	3.07 (0.93)	2.99 (1.09)	3.61**	0.036	1 < 3
Self-Assurance	3.04 (0.88)	3.24 (0.95)	3.47 (0.86)	3.46 (0.92)	3.10*	0.031	1 < 3
Attentiveness	3.14 (0.79)	3.31 (0.84)	3.59 (0.80)	3.49 (0.93)	3.92**	0.039	1 < 3
Fear	2.59 (0.82)	2.22 (0.79)	2.23 (0.84)	2.16 (0.94)	2.54	0.026	–
Self-Disgust	1.73 (0.62)	1.65 (0.73)	1.64 (0.64)	1.52 (0.62)	0.95	0.010	–
Hostility	2.56 (1.16)	2.06 (1.24)	1.97 (1.03)	2.01 (1.11)	3.26*	0.033	1 > 3

**p < 0.01, *p < 0.05.

There were sex differences in the level of participation [χ^(2)^ = 31.48, *p* < 0.001], with males more often belonging to the group of elite athletes and being more physically active. Additionally, there were also age differences in the level of sport participation with elite athletes were older [*F*(2,394) = 4.24, *p* < 0.05]. In preliminary analysis, statistically controlled sex and age did not have any influence to the obtained effects, thus they are not included as control variables.

Data were collected online via social networks, including groups and websites of recreation and sports associations. Data were collected in the period from April 20th till April 30th (during the 6th and the 7th week of the emergency state and curfew). The study was approved by the Ethical Committee of the Faculty of Philosophy, University of Novi Sad, Serbia, which is the Second Instance Commission of the Ethical Committee of the Serbian Psychological Society (Code: 202004092113_Gfu4).

#### Measures

The Satisfaction With Life Scale (SWLS; Diener et al., 1985, for the Serbian adaptation see Vasić et al., 2011) comprises five items measuring the cognitive domain of subjective well-being. The instruction was to rate items on a 7-point scale (from 1 = *strongly disagree* to 7 = *strongly agree*) relative to the general state, before the pandemic and the emergency state (α = 0.91), and the previous week, while the emergency state and curfew lasted (α = 0.86).

The Positive and Negative Affect Schedule (PANAS; Watson et al., 1988, for the Serbian adaptation see Mihic̀ et al., 2014) consists of 20 items measuring the affective domain of well-being, i.e., positive and negative affect, with 10 items tapping each. Positive affect includes subscales of joviality (α_before_ = 0.79, α_curfew_ = 0.83), self-assurance (α_before_ = 0.81, α_curfew_ = 0.77), and attentiveness (α_before_ = 0.75, α_curfew_ = 0.78), while negative affect includes subscales of fear (α_before_ = 0.89, α_curfew_ = 0.86), self-disgust (α_before_ = 0.69, α_curfew_ = 0.53), and hostility (α_before_ = 0.75, α_curfew_ = 0.72). The instruction was to rate items on a 5-point scale (from 1 = *very slightly or not at all* to 5 = *extremely*), again, relative to the period before the pandemic and the emergency state and to the previous week, while emergency state and curfew lasted.

Two multiple-choice questions were added in order to assess the changes in the routines of physical activity due to the curfew (“*How often did you use to train before the state of emergency was declared*?” and “*How often have you been training since the state of emergency was declared?*”).

This study is a part of a larger project which includes other measures, but all the measures that refer to the state of emergency were given at the beginning of the instruments set, and these were followed by the measures referring to the period before the state of emergency.

### Results

**FIGURE 2:**
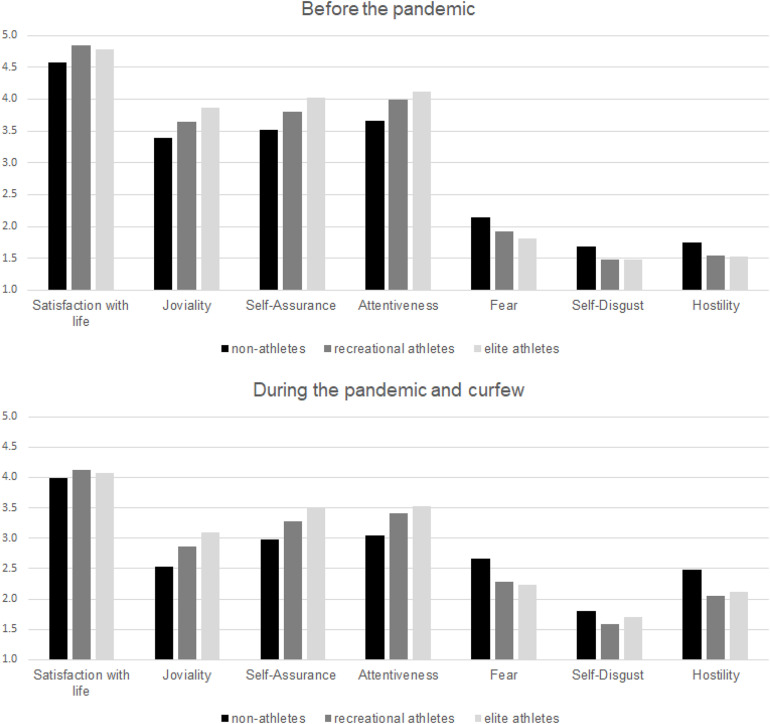
Well-being domains before **(top)** and during the emergency state and curfew **(bottom)** in non-athletes, recreational athletes, and elite athletes (*N* = 398).

Firstly, mixed-design ANOVAs with one repeated-measure factor (before and during the curfew) and one between-subject factor (groups based on sports participation) were conducted, for each indicator of well-being as a dependent variable. The results showed a significant decrease in well-being during the emergency state in all groups (Table 6 and Figure 2). There was also the main effect of the group on positive and negative affect, with non-athletes showing lower positive affect and higher negative affect, compared to recreational and elite athletes who were not mutually different. Thus, as for non-athletes, this tendency is independent of the estimation period. However, there were no significant interactions between the repeated-measure factor and the between-subject factor (*F*s ranged between 0.23 and 0.50, all *p* > 0.05), meaning that belonging to a group based on sports participation did not change the decrease in well-being.

**TABLE 6:** Results of mixed ANOVAs: differences in well-being before and during the curfew between non-athletes, recreational athletes, and elite athletes (*N* = 398).

**Well-being**	**Repeated-measures factor**		**Between-subject factor**		
	**(before and during curfew)**		**(sport participation)**		
	***F*(1,395)**	**η^2^**	***F*(2,395)**	**η_*p*_^2^**	***Post hoc***
Satisfaction with life	148.23***	0.273	0.99	0.005	–
Joviality	221.07***	0.359	15.44***	0.072	1 < 2 < 3
Self-Assurance	118.39***	0.231	14.66***	0.069	1 < 2 < 3
Attentiveness	166.99***	0.297	16.94***	0.079	1 < 2, 3
Fear	83.07***	0.174	8.68***	0.042	1 > 2, 3
Self-Disgust	18.29***	0.044	4.47**	0.022	1 > 2
Hostility	128.07***	0.245	5.49**	0.027	1 > 2, 3

1 = non-athletes (103), 2 = recreational athletes (180), 3 = elite athletes (115), ***p < 0.001, **p < 0.01.

Secondly, two-way ANOVAs with sports participation (recreational and elite athletes) and changes in the training routine as factors was conducted for each indicator of well-being as a dependent variable. However, since interactions between the two factors were not significant (*F*s ranged from 0.35 to 1.40, all *p* > 0.05), only the effects of the training routine were calculated. Results of the one-way ANOVAs showed that changes in the training routine among athletes had a significant effect on all the facets of positive affect as well as on hostility, with athletes who became inactive reporting lower positive affect and higher hostility during the curfew compared to those who kept the same training routine (Table 5).

In sum, we confirmed that well-being decreased among all the participants during the curfew (H1) and that recreational and elite athletes had better well-being both before and during the curfew compared to non-athletes (H2). However, we did not confirm that decrease in well-being is smaller among recreational and elite athletes (H3). Thus, being physically active did not provide additional benefits for mental health in crises and stress situations, but rather reflected the same benefits as before the pandemic and the curfew. Finally, we confirmed that the athletes who did not change their training routine during the curfew show better well-being compared to those who became inactive (H4), but there is no difference in regard to those who reduced or increased their trainings.

## General Discussion

Since the beginning of the pandemic, numerous studies have been published regarding the importance of physical activity in the general population during lockdown. WHO recommendations (WHO, 2020b) specifically include periodic times of moderate-intensity or vigorous-intensity physical activity during the week, which have proven to be particularly helpful, especially in times of anxiety, crisis, and fear (e.g., Aldana et al., 1996; Chekroud et al., 2018). These recommendations for the general population are mostly based on the idea that the beneficial effects of physical activity on mental health include distraction, self-efficacy, and social interaction (Peluso and Andrade, 2005). However, in the context of the lockdown and curfew, there are concerns that the reduced (or even complete lack of) access to regular routines may result in mental health disruptions in individuals who engage in sports or exercise. As athletes represent a highly unique cohort, the general WHO recommendations on physical activity in “at-home conditions” do not meet their sport-specific requirements (e.g., maximal musculoskeletal tension during specific movement such as sprinting). Although there have been some insights into physiological and musculoskeletal consequences of long-term detraining (Mujika and Padilla, 2000), little is known about the psychological aftermath of such forced and lingering circumstances.

In the present paper, we aimed to provide a comprehensive insight into the effects of different physical activity factors on mental health during different stages of the emergency state and curfew. Regarding differences in mental health indicators among non-athletes, recreational athletes, and elite athletes, the results of Study 1 showed that elite athletes, followed by the recreational athletes, had less psychological distress compared to the physically inactive (non-athletes). Although those who are physically active report less distress, contrary to our expectation, elite athletes showed significantly lower distress levels compared to recreational athletes. Although the group of elite athletes in our study is comprised of those that are mostly involved in national competitions, results are in line with a recent study that showed no impact of the pandemic on the anxiety response of Olympic and Paralympic athletes in the first wave of pandemic (Clemente-Suárez et al., 2020). These results are in line with some previous studies (e.g., McAllister et al., 2001; Modolo et al., 2009; Shirvani et al., 2015) and showed that elite athletes could react more adaptively to stressful situations and crises. It could be assumed that chronic exposure to pressures helps elite athletes strengthen their capacity to overcome novel stressful and challenging situations, including a situation such as a crisis caused by a pandemic.

Study 2 on another sample further provides evidence of better well-being, or more precisely its affective domain, among physically active individuals. At the global level, elite athletes had higher positive affect and both elite and recreational athletes had lower negative affect compared to non-athletes. Therefore, we could conclude that athletes, especially elite athletes, had better mental health in general, compared to non-athletes.

Study 2 revealed that the affective domain of well-being was reduced during the curfew in all participants. This is in line with studies that have revealed an overall decrease in subjective well-being during the early stages of the COVID-19 pandemic (e.g., Zacher and Rudolph, 2020), with negative affect decreasing over time (e.g., Sadiković et al., 2020). However, level of sports participation did not affect the decrease of emotional aspect of well-being. This means that although athletes have better mental health, the global crisis situation resulting from the current pandemic has negatively affected everyone and engagement in physical activity has failed to moderate the decrease in mental health indicators. Thus, being physically active did not provide additional benefits for mental health, but rather reflect the same benefit that exists in non-crisis situations. However, since elite athletes have been found to have better mental health, this could make them more resilient to stressful and crisis situations compared to recreational athletes or non-athletes. It seems that elite athletes have some kind of “reservoir” of energy and self-assurance, which could lead to better adjustment even if mental health is lower overall.

Interestingly, there were no differences between athletes and non-athletes in the cognitive domain of well-being, but only in the affective domain. The distinction between cognitive and affective components of well-being has been well-documented (e.g., Luhmann et al., 2012). Previous studies have shown inconsistent results regarding differences in life satisfaction between athletes and non-athletes (e.g., Norinejad et al., 2014; Ivantchev and Stoyanova, 2019). One of the explanations could be that life satisfaction is a more stable component of well-being compared to the affective component. Thus, physical activity may have greater influence on mood changes (e.g., López-Bueno et al., 2020). Additionally, the cognitive domain is more related to the basic psychological needs (e.g., food or salary), while the affective domain is more related to social exchange and close relationships (Diener et al., 2009). Therefore, we could assume that athletes have extra social support, including members of their teams or sports groups.

When considering the level of intensity of physical activity, in the context of responding to the early stage of the curfew in Study 1, our results showed that participants with previous vigorous activity scored lowest on all dimensions of psychological distress compared to those who were previously moderately active or sedentary. Since all elite athletes belonged to the group of vigorous activity, this result is in line with previously mentioned findings (e.g., Shirvani et al., 2015). However, the group of vigorous activity was the most numerous and also included recreational athletes as well as those who considered themselves as non-athletes, although the latter were small in number. Besides the explanation referring to better mental health among those who were highly physically active, there is an alternative explanation of an effect on anxiety. The effect on anxiety could be explained by taking into account the physiological component of anxiety. Namely, it is possible that individuals who engage in vigorous physical activity are less sensitive or are used to physiological changes, e.g., increased heart and breathing rate, sweating or a rise in body temperature, all of which are inherent to anxiety. This complements previous findings that regular exercise might facilitate habituation and higher tolerance to the sensations of anxiety (Broman-Fulks and Storey, 2008; Ströhle et al., 2009, although not taking into account the intensity of physical activity directly). For further implications, a more detailed inspection of the specific time periods of intense activity engagement is needed.

Considering the effect of the training routine on mental health, the results of the two studies showed seemingly conflicting results. In Study 1, results showed that the effect of changes in the training routine on distress depends on the level of sports participation. Thus, elite athletes who reduced the training routine showed lower anxiety compared to recreational athletes who also reduced or kept the same training routine during the early stage of the curfew. Additionally, recreational athletes who reduced their trainings showed lower anxiety compared to those who kept the same training routine. Having in mind that Study 1 was conducted in the early stage of the curfew, it could be assumed that keeping the same routine requires extra effort and perhaps rescheduling other activities due to the limited time that could be spent outside. Being a recreational athlete usually means that the one is engaged in physical activity in leisure time, after work or other obligations. Since the curfew limits leisure time that recreational athletes usually spend outside or in sports objects that are now closed due to the pandemic, maintaining the training routine could be a challenging task. Thus, trying to balance the training routine and compliance with governmentally enforced restrictions related to COVID-19 could have resulted in additional stress, which had a negative effect on mental health among recreational athletes who were trying to keep the same training routine.

Alternative explanation could be that the group of recreational athletes might include a number of those prone to engaging in maladaptive coping strategies, e.g., failing to accept novel circumstances, engaging in avoidant behaviors, and trying to keep control over the situation by not changing the routine, or those prone to overtraining, giving overlapping symptoms with depression (e.g., low self-confidence, lack of appetite) and stress (e.g., sleep disturbances, irritability). It should be noted that the recreational athletes who kept the same training routine at this stage constituted a minority of physically active participants (55), while the majority (317) reduced their trainings.

Compared to recreational athletes, elite athletes who reduced trainings showed lower levels of anxiety. There are highly important implications of this result, coupled with the evidence that properly reduced training can be enough for achieving previous physical performance later on (Huyghe et al., 2020). Our results suggest that during the early stages of the curfew, when all resources were focused on adapting to the novel stressful situation and life conditions, elite athletes showed more adaptability to the novel circumstances and the curfew. Our results are in line with a conclusion of Huyghe et al. (2020) that athletes could benefit from focusing on the improvement of non-physical aspects of sports performance, while keeping the “minimum effective dose” in training. The period of adjustment and adaptation to this kind of unique stressor is necessary, until conditions are met for recreating one’s daily routine.

However, in the later stage as Study 2 showed, those who kept the same training routine showed better affective domain of well-being compared to those who became inactive. In the later stage of the curfew, almost half of the physically active individuals (48.5%) managed to keep the same training routine and there were also those who even increased trainings (19.7%). Thus, it could be assumed that after some time of adjustment to novel circumstances and the restoration of a daily routine, those who continued with trainings in line with their routine before the pandemic showed better mental health. Thus, there is no additional benefit of being an elite athlete and keeping the same training routine that contributes to better well-being.

There are several limitations of this research. First, although in Study 2, the measures were given in two instructions (before and during the pandemic and the emergency state), reports of mental health before the pandemic rely on memory. Although all participants first filled out measures in line with the current state and then in line with the state before the pandemic, it is possible that they emphasized the negative effects of the current pandemic situation. Future studies should consider a longitudinal design in order to address the changes in mental health during the pandemic. Second, although the same recruitment method was used, the two studies were conducted on different samples. Thus, a comparison between the results of these two samples should be made with caution. Third, the differentiation between elite athletes, recreational athletes, and non-athletes was based on self-report. In Study 2, other criteria were used. However, as we could see in Study 1, some highly physically active participants may consider themselves as non-athletes. Thus, future studies should include some other, objective criteria for differentiating between these groups, i.e., sports and recreational club memberships in combination with a training routine and participation in competitions. Finally, although in preliminary analysis statistically controlled sex and age did not have any influence to the obtained effects, future studies should include more female elite athletes and both junior and senior athletes in order to test their possible moderation effect.

As our results contradict the evidence of better mental health among recreational and/or moderately active athletes, it might be that the “social outlet” (Schaal et al., 2011) aspect of physical activity is an important factor to consider within this group. There are hints that some benefits of physical activity rely greatly on social contact, as could be seen in recreational athletes (Zarrett et al., 2015). Given the context of social distancing, this should be further inspected.

In sum, we found that elite athletes, as well as individuals engaging in vigorous physical activity, showed the highest ability to adapt to the current crisis. More importantly, this result suggests that although in terms of mental health, the adequate first response to a crisis might require some adjustments in the daily routine (training-wise), keeping and adapting previous routines to new circumstances leads to long-term mental health benefits.

## Data Availability Statement

The raw data supporting the conclusions of this article will be made available by the authors, without undue reservation.

## Ethics Statement

The studies involving human participants were reviewed and approved by Study 1: Ethical Committee of the Faculty of Sport and Tourism, Educons University, Novi Sad, Serbia; Study 2: Ethical Committee of the Faculty of Philosophy, University of Novi Sad, Serbia, which is the Second Instance Commission of the Ethical Committee of the Serbian Psychological Society (Code: 202004092113_Gfu4). The patients/participants provided their written informed consent to participate in this study.

## Author Contributions

JS and SP conducted the Study 1. BD and JR conducted the Study 2. JS and BD wrote the original and revised manuscript and performed the analysis of the results, with inputs from SP and JR. All authors contributed to the design and implementation of the two conducted studies, discussed the results, commented on the manuscript, and contributed to the final version of the manuscript.

## Conflict of Interest

The authors declare that the research was conducted in the absence of any commercial or financial relationships that could be construed as a potential conflict of interest.

## Footnotes

1 http://www2.psy.unsw.edu.au/dass/Serbian/DASS-SER.pdf

## References

[B1] Aldana S. G. Sutton L. D. Jacobson B. H. Quirk M. G. (1996). Relationships between leisure time physical activity and perceived stress. *Percept. Mot. Skills* 82 315–321. 10.2466/pms.1996.82.1.315 8668498

[B2] Asztalos M. Wijndaele K. De Bourdeaudhuij I. Philippaerts R. Matton L. Duvigneaud N. (2012). Sport participation and stress among women and men. *Psychol. Sport Exerc.* 13 466–483. 10.1016/j.psychsport.2012.01.003

[B3] Belinchón-deMiguel P. Ruisoto-Palomera P. Clemente-Suárez V. J. (2019). Psychophysiological stress response of a Paralympic athlete during an ultra–endurance event. A case study. *J. Med. Syst.* 43:70. 10.1007/s10916-019-1188-6 30737600

[B4] Broman-Fulks J. J. Storey K. M. (2008). Evaluation of a brief aerobic exercise intervention for high anxiety sensitivity. *Anxiety Stress Coping* 21 117–128. 10.1080/10615800701762675 18350391

[B5] Bull F. C. Al-Ansari S. S. Biddle S. Borodulin K. Buman M. P. Cardon G. (2020). World Health Organization 2020 guidelines on physical activity and sedentary behaviour. *Br. J. Sports Med.* 54 1451–1462. 10.1136/bjsports-2020-102955 33239350 PMC7719906

[B6] Callaghan P. (2004). Exercise: a neglected intervention in mental health care? *J. Psychiatr. Ment. Health Nurs.* 11 476–483. 10.1111/j.1365-2850.2004.00751.x 15255923

[B7] Castillo I. Molina-García J. Álvarez O. (2010). Importance of perceived competition and motivation to the mental health of college athletes. *Salud Publica Mex.* 52 517–523. 21271010

[B8] Chekroud S. R. Gueorguieva R. Zheutlin A. B. Paulus M. Krumholz H. M. Krystal J. H. (2018). Association between physical exercise and mental health in 1.2 million individuals in the USA between 2011 and 2015: a cross–sectional study. *Lancet Psychiatry* 5 739–746. 10.1016/s2215-0366(18)30227-x 30099000

[B9] Clemente-Suárez V. J. Fuentes-García J. P. de la Vega Marcos R. Patiño M. J. M. (2020). Modulators of the personal and professional threat perception of Olympic athletes in the actual COVID–19 crisis. *Front. Psychol.* 11:1985. 10.3389/fpsyg.2020.01985 32849157 PMC7419607

[B10] Connaughton D. Wadey R. Hanton S. Jones G. (2008). The development and maintenance of mental toughness: perceptions of elite performers. *J. Sports Sci.* 26 83–95. 10.1080/02640410701310958 17852671

[B11] Costigan S. A. Lubans D. R. Lonsdale C. Sanders T. del Pozo Cruz B. (2019). Associations between physical activity intensity and well–being in adolescents. *Prev. Med.* 125 55–61. 10.1016/j.ypmed.2019.05.009 31125627

[B12] Cresswell S. L. Eklund R. C. (2007). Athlete burnout: a longitudinal qualitative study. *Sport Psychol.* 21 1–20. 10.1123/tsp.21.1.1

[B13] Diener E. Lucas R. Schimmack U. Helliwell J. (2009). *Well–Being for Public Policy.* New York, NY: Oxford University Press, 10.1093/acprof:oso/9780195334074.001.0001

[B14] Diener E. D. Emmons R. A. Larsen R. J. Griffin S. (1985). The satisfaction with life scale. *J. Pers. Assess.* 49 71–75. 10.1207/s15327752jpa4901_13 16367493

[B15] Downward P. Dawson P. (2016). Is it pleasure or health from leisure that we benefit from most? An analysis of well–being alternatives and implications for policy. *Soc. Indic. Res.* 126 443–465. 10.1007/s11205-015-0887-8

[B16] Ekkekakis P. (2015). Honey, I shrunk the pooled SMD! Guide to critical appraisal of systematic reviews and meta–analyses using the Cochrane review on exercise for depression as example. *Ment. Health Phys. Act.* 8 21–36. 10.1016/j.mhpa.2014.12.001

[B17] Frontini R. Rebelo-Gonçalves R. Amaro N. Salvador R. Matos R. Morouço P. (2021). The relationship between anxiety levels, sleep, and physical activity during COVID-19 lockdown: an exploratory study. *Front. Psychol.* 12:659599. 10.3389/fpsyg.2021.659599 33859601 PMC8042226

[B18] Galea S. Merchant R. M. Lurie N. (2020). The mental health consequences of COVID-19 and physical distancing: the need for prevention and early intervention. *JAMA Intern. Med.* 180 817–818. 10.1001/jamainternmed.2020.1562 32275292

[B19] Gerber M. Holsboer-Trachsler E. Pühse U. Brand S. (2011). Elite sport is not an additional source of distress for adolescents with high stress levels. *Percept. Mot. Skills* 112 581–599. 10.2466/02.05.10.pms.112.2.581-599 21667766

[B20] Godin G. Shephard R. J. (1985). A simple method to assess exercise behavior in the community. *Can. J. Appl. Sport Sci.* 10 141–146. 10.14288/hfjc.v4i1.82 4053261

[B21] Gouttebarge V. Backx F. J. Aoki H. Kerkhoffs G. M. (2015). Symptoms of common mental disorders in professional football (soccer) across five European countries. *J. Sports Sci. Med.* 14 811–818. 10.1080/00913847.2017.1248796 26664278 PMC4657424

[B22] Gulliver A. Griffiths K. M. Mackinnon A. Batterham P. J. Stanimirovic R. (2015). The mental health of Australian elite athletes. *J. Sci. Med. Sport* 18 255–261. 10.1016/j.jsams.2014.04.006 24882147

[B23] Harvey S. B. Øverland S. Hatch S. L. Wessely S. Mykletun A. Hotopf M. (2018). Exercise and the prevention of depression: results of the HUNT cohort study. *Am. J. Psychiatry* 175 28–36. 10.1176/appi.ajp.2017.16111223 28969440

[B24] Hughes L. Leavey G. (2012). Setting the bar: athletes and vulnerability to mental illness. *Br. J. Psychiatry* 200 95–96. 10.1192/bjp.bp.111.095976 22297587

[B25] Huyghe T. G. Bird S. P. Calleja-González J. Alcaraz P. E. (2020). Season suspension and summer extension: unique opportunity for professional team–sport athletes and support staff during and following the COVID–19 crisis. *Front. Sports Act. Living* 2:98. 10.3389/fspor.2020.00098 33345088 PMC7739718

[B26] Ivantchev N. Stoyanova S. (2019). Athletes and Non–Athletes’ Life Satisfaction. *Athens J. Sports* 6 45–60. 10.30958/ajspo.6-1-4

[B27] John A. Pirkis J. Gunnell D. Appleby L. Morrissey J. (2020). Trends in suicide during the covid-19 pandemic. *BMJ* 371:m4352. 10.1136/bmj.m4352 33184048

[B28] Jonsdottir H. I Rödjer L. Hadzibajramovic E. Börjesson M. Ahlborg G. Jr. (2010). A prospective study of leisure–time physical activity and mental health in Swedish health care workers and social insurance officers. *Prev. Med.* 51 373–377. 10.1016/j.ypmed.2010.07.019 20691721

[B29] Jovanovic̀ V. Gavrilov-Jerkovic̀ V. Žuljevic̀ D. Brdaric̀ D. (2014). Psihometrijska evaluacija Skale depresivnosti, anksioznosti i stresa-21 (DASS-21) na uzorku studenata u Srbiji (Psychometric evaluation of the Depression Anxiety Stress Scales–21 (DASS–21) in a Serbian student sample). *Psihologija* 47 93–112. 10.2298/PSI1401093J

[B30] Lawlor D. A. Hopker S. W. (2001). The effectiveness of exercise as an intervention in the management of depression: systematic review and meta–regression analysis of randomised controlled trials. *BMJ* 322 763–767. 10.1136/bmj.322.7289.763 11282860 PMC30551

[B31] Liu X. Kakade M. Fuller C. J. Fan B. Fang Y. Kong J. (2012). Depression after exposure to stressful events: lessons learned from the severe acute respiratory syndrome epidemic. *Compr. Psychiatry* 53 15–23. 10.1016/j.comppsych.2011.02.003 21489421 PMC3176950

[B32] López-Bueno R. Calatayud J. Ezzatvar Y. Casajús J. A. Smith L. Andersen L. L. (2020). Association between current physical activity and current perceived anxiety and mood in the initial phase of COVID–19 confinement. *Front. Psychiatry* 11:729. 10.3389/fpsyt.2020.00729 32793013 PMC7390883

[B33] Lovibond P. F. Lovibond S. H. (1995). The structure of negative emotional states: comparison of the depression anxiety stress scales (DASS) with the Beck Depression and Anxiety Inventories. *Behav. Res. Ther.* 33 335–343. 10.1016/0005-7967(94)00075-u 7726811

[B34] Luhmann M. Hawkley L. C. Eid M. Cacioppo J. T. (2012). Time frames and the distinction between affective and cognitive well–being. *J. Res. Pers.* 46 431–441. 10.1016/j.jrp.2012.04.004 23420604 PMC3571101

[B35] Maugeri G. Castrogiovanni P. Battaglia G. Pippi R. D’Agata V. Palma A. (2020). The impact of physical activity on psychological health during Covid–19 pandemic in Italy. *Heliyon* 6:e04315. 10.1016/j.heliyon.2020.e04315 32613133 PMC7311901

[B36] McAllister D. R. Motamedi A. R. Hame S. L. Shapiro M. S. Dorey F. J. (2001). Quality of life assessment in elite collegiate athletes. *Am. J. Sports Med.* 29 806–810. 10.1177/03635465010290062201 11734497

[B37] Mihic̀ L. Novovic̀ Z. Èolovic̀ P. Smederevac S. (2014). Serbian adaptation of the Positive and Negative Affect Schedule (PANAS): its facets and second–order structure. *Psihologija* 47 393–414. 10.2298/PSI1404393M

[B38] Modolo V. B. Mello M. T. Gimenez P. R. B. Tufik S. Antunes H. K. M. (2009). Physical exercise dependence: mood, quality of life in amateur and professional athletes. *Braz. J. Sport Med.* 15 355–359.

[B39] Morres I. D. Hatzigeorgiadis A. Krommidas C. Comoutos N. Sideri E. Ploumpidis D. (2019). Objectively measured physical activity and depressive symptoms in adult outpatients diagnosed with major depression. Clinical perspectives. *Psychiatry Res.* 280:112489. 10.1016/j.psychres.2019.112489 31442671

[B40] Mujika I. Padilla S. (2000). Detraining: loss of training–induced physiological and performance adaptations. Part II: long term insufficient training stimulus. *Sports Med.* 30 145–154. 10.2165/00007256-200030030-00001 10999420

[B41] Netz Y. Wu M. J. Becker B. J. Tenenbaum G. (2005). Physical activity and psychological well–being in advanced age: a meta–analysis of intervention studies. *Psychol. Aging* 20 272–284. 10.1037/0882-7974.20.2.272 16029091

[B42] Nixdorf I. Frank R. Beckmann J. (2016). Comparison of athletes’ proneness to depressive symptoms in individual and team sports: research on psychological mediators in junior elite athletes. *Front. Psychol.* 7:893. 10.3389/fpsyg.2016.00893 27378988 PMC4911498

[B43] Norinejad H. Naghiloo Z. Soroushnia R. Dezhahang M. Kavandi H. (2014). Comparing general health and life satisfaction among athlete versus non-athlete students in Islamic Azad University, Hidaj. *Indian J. Fundam. Appl. Life Sci.* 4 2058–2063.

[B44] Oztekin H. H. Boya H. Ozcan O. Zeren B. Pinar P. (2008). Pain and affective distress before and after ACL surgery: a comparison of amateur and professional male soccer players in the early postoperative period. *Knee* 15 368–372. 10.1016/j.knee.2008.05.007 18635361

[B45] Panza G. A. Taylor B. A. Thompson P. D. White C. M. Pescatello L. S. (2019). Physical activity intensity and subjective well–being in healthy adults. *J. Health Psychol.* 24 1257–1267. 10.1177/1359105317691589 28810402

[B46] Pappa S. Ntella V. Giannakas T. Giannakoulis V. G. Papoutsi E. Katsaounou P. (2020). Prevalence of depression, anxiety, and insomnia among healthcare workers during the COVID–19 pandemic: a systematic review and meta–analysis. *Brain Behav. Immun.* 88 901–907. 10.1016/j.bbi.2020.05.026 32437915 PMC7206431

[B47] Peluso M. A. M. Andrade L. H. S. G. D. (2005). Physical activity and mental health: the association between exercise and mood. *Clinics* 60 61–70. 10.1590/s1807-59322005000100012 15838583

[B48] Pinc̀ir T. Crvenko A. Sokic̀ J. Popov S. (2020). Do physically active individuals experience more satisfying sex? Manuscript in preparation.

[B49] Popov S. Sokic̀ J. Stupar D. (2021). Activity matters: physical exercise and stress coping during the 2020 COVID-19 state of emergency. *Psihologija.* 10.2298/PSI200804002P

[B50] Putukian M. (2016). The psychological response to injury in student athletes: a narrative review with a focus on mental health. *Br. J. Sports Med.* 50 145–148. 10.1136/bjsports-2015-095586 26719498

[B51] Rajkumar R. P. (2020). COVID–19 and mental health: a review of the existing literature. *Asian J. Psychiatry* 52:102066. 10.1016/j.ajp.2020.102066 32302935 PMC7151415

[B52] Reardon C. L. Factor R. M. (2010). Sport psychiatry: a systematic review of diagnosis and medical treatment of mental illness in athletes. *Sports Med.* 40 961–980. 10.2165/11536580-000000000-00000 20942511

[B53] Reardon C. L. Hainline B. Aron C. M. Baron D. Baum A. L. Bindra A. (2019). Mental health in elite athletes: International Olympic Committee consensus statement (2019). *Br. J. Sports Med.* 53 667–699. 10.1136/bjsports-2019-100715 31097450

[B54] Rice S. M. Purcell R. De Silva S. Mawren D. McGorry P. D. Parker A. G. (2016). The mental health of elite athletes: a narrative systematic review. *Sports Med.* 46 1333–1353. 10.1007/s40279-016-0492-2 26896951 PMC4996886

[B55] Sadiković S. Branovaèki B. Oljaèa M. Mitrovic̀ D. Pajic̀ D. Smederevac S. (2020). Daily monitoring of emotional responses to the coronavirus pandemic in Serbia: a citizen science approach. *Front. Psychol.* 11:2133. 10.3389/fpsyg.2020.02133 32973636 PMC7466566

[B56] Schaal K. Tafflet M. Nassif H. Thibault V. Pichard C. Alcotte M. (2011). Psychological balance in high level athletes: gender–based differences and sport–specific patterns. *PLoS One* 6:e19007. 10.1371/journal.pone.0019007 21573222 PMC3087722

[B57] Shirvani H. Barabari A. Keshavarz Afshar H. (2015). A comparison of cognitive emotion regulation strategies in semi professional and amateur athletes. *J. Mil. Med.* 16 237–242.

[B58] Sprang G. Silman M. (2013). Posttraumatic stress disorder in parents and youth after health-related disasters. *Disaster Med. Public Health Prep.* 7 105–110. 10.1017/dmp.2013.22 24618142

[B59] Stašević-Karličić I. Ðorđević V. Stašević M. Subotic̀ T. Filipovic̀ Z. Ignjatovic̀-Ristic̀ D. (2020). Perspectives on mental health services during the COVID-19 epidemic in Serbia. *Srp. Arh. Celok. Lek.* 148 28–28. 10.2298/SARH200504028S

[B60] Steptoe A. Butler N. (1996). Sports participation and emotional wellbeing in adolescents. *Lancet* 347 1789–1792. 10.1016/s0140-6736(96)91616-5 8667922

[B61] Ströhle A. Graetz B. Scheel M. Wittmann A. Feller C. Heinz A. (2009). The acute antipanic and anxiolytic activity of aerobic exercise in patients with panic disorder and healthy control subjects. *J. Psychiatr. Res.* 43 1013–1017. 10.1016/j.jpsychires.2009.02.004 19289240

[B62] Stubbs B. Koyanagi A. Hallgren M. Firth J. Richards J. Schuch F. (2017a). Physical activity and anxiety: a perspective from the World Health Survey. *J. Affect. Disord.* 208 545–552. 10.1016/j.jad.2016.10.028 27802893

[B63] Stubbs B. Vancampfort D. Rosenbaum S. Firth J. Cosco T. Veronese N. (2017b). An examination of the anxiolytic effects of exercise for people with anxiety and stress–related disorders: a meta–analysis. *Psychiatry Res.* 249 102–108. 10.1016/j.psychres.2016.12.020 28088704

[B64] Taylor A. H. (2000). “Physical activity, anxiety, and stress,” in *Physical Activity and Psychological Well–Being*, eds Biddle S. J. Fox K. Boutcher S. (London: Routledge), 10–45.

[B65] Timperio A. Salmon J. Rosenberg M. Bull F. C. (2004). Do logbooks influence recall of physical activity in validation studies? *Med. Sci. Sports Exerc.* 36 1181–1186. 10.1249/01.mss.0000132268.74992.d8 15235322

[B66] Vasić A. Šarèevic̀ D. Trogrlic̀ A. (2011). Zadovoljstvo životom u Srbiji [Satisfaction with life in Serbia]. *Primenjena Psihol.* 4 151–177. 10.19090/pp.2011.2.151-177

[B67] Wang C. Pan R. Wan X. Tan Y. Xu L. Ho C. S. (2020). Immediate psychological responses and associated factors during the initial stage of the 2019 coronavirus disease (COVID-19) epidemic among the general population in China. *Int. J. Environ. Res. Public Health* 17:1729. 10.3390/ijerph17051729 32155789 PMC7084952

[B68] Watson D. Clark L. A. Tellegen A. (1988). Development and validation of brief measure of Positive and Negative Affect: the PANAS scales. *J. Pers. Soc. Psychol.* 54 1063–1070. 10.1037/0022-3514.54.6.1063 3397865

[B69] White R. L. Babic M. J. Parker P. D. Lubans D. R. Astell-Burt T. Lonsdale C. (2017). Domain–specific physical activity and mental health: a meta–analysis. *Am. J. Prev. Med.* 52 653–666. 10.1016/j.amepre.2016.12.008 28153647

[B70] WHO (2018). Mental Health: Strengthening Our Response. Fact Sheet. Available online at: http://www.who.int/en/news-room/fact-sheets/detail/mental-health-strengthening-our-response

[B71] WHO (2020a). *WHO Coronavirus Disease (COVID–19) Dashboard.* Available online at: https://www.who.int/emergencies/diseases/novel–coronavirus–2019 (accessed September 26, 2020).

[B72] WHO (2020b). *#HealthyAtHome – Physical Activity.* Available online at: https://www.who.int/news–room/campaigns/connecting–the–world–to–combat–coronavirus/healthyathome/healthyathome——physical–activity 2020b (accessed March 27, 2020).

[B73] Wicker P. Frick B. (2015). The relationship between intensity and duration of physical activity and subjective well–being. *Eur. J. Public Health* 25 868–872. 10.1093/eurpub/ckv131 26142405

[B74] Zacher H. Rudolph C. W. (2020). Individual differences and changes in subjective wellbeing during the early stages of the COVID–19 pandemic. *Am. Psychol.* 76 50–62. 10.1037/amp0000702 32700938

[B75] Zarrett N. Sorensen C. Cook B. S. (2015). Physical and social–motivational contextual correlates of youth physical activity in underresourced afterschool programs. *Health Educ. Behav.* 42 518–529. 10.1177/1090198114564502 25588937

[B76] Zhang S. X. Wang Y. Rauch A. Wei F. (2020). Unprecedented disruptions of lives and work: health, distress and life satisfaction of working adults in China one month into the COVID–19 outbreak. *Psychiatry Res.* 288:112958. 10.1016/j.psychres.2020.112958 32283450 PMC7146665

